# SARS-CoV-2 vaccine ChAdOx1 nCoV-19 infection of human cell lines reveals low levels of viral backbone gene transcription alongside very high levels of SARS-CoV-2 S glycoprotein gene transcription

**DOI:** 10.1186/s13073-021-00859-1

**Published:** 2021-03-15

**Authors:** Abdulaziz Almuqrin, Andrew D. Davidson, Maia Kavanagh Williamson, Philip A. Lewis, Kate J. Heesom, Susan Morris, Sarah C. Gilbert, David A. Matthews

**Affiliations:** 1 School of Cellular and Molecular Medicine, Faculty of Life Sciences, University Walk, University of Bristol, Bristol, BS8 1TD UK; 2 Department of Clinical Laboratory Sciences, King Saud University, Riyadh, Saudi Arabia; 3 Proteomics Facility, Faculty of Life Sciences, University Walk, University of Bristol, Bristol, BS8 1TD UK; 4 Jenner Institute, Nuffield Department of Medicine, University of Oxford, Oxford, OX3 7DQ UK

## Abstract

**Background:**

ChAdOx1 nCoV-19 is a recombinant adenovirus vaccine against SARS-CoV-2 that has passed phase III clinical trials and is now in use across the globe. Although replication-defective in normal cells, 28 kbp of adenovirus genes is delivered to the cell nucleus alongside the SARS-CoV-2 S glycoprotein gene.

**Methods:**

We used direct RNA sequencing to analyse transcript expression from the ChAdOx1 nCoV-19 genome in human MRC-5 and A549 cell lines that are non-permissive for vector replication alongside the replication permissive cell line, HEK293. In addition, we used quantitative proteomics to study over time the proteome and phosphoproteome of A549 and MRC5 cells infected with the ChAdOx1 nCoV-19 vaccine.

**Results:**

The expected SARS-CoV-2 S coding transcript dominated in all cell lines. We also detected rare S transcripts with aberrant splice patterns or polyadenylation site usage. Adenovirus vector transcripts were almost absent in MRC-5 cells, but in A549 cells, there was a broader repertoire of adenoviral gene expression at very low levels. Proteomically, in addition to S glycoprotein, we detected multiple adenovirus proteins in A549 cells compared to just one in MRC5 cells.

**Conclusions:**

Overall, the ChAdOx1 nCoV-19 vaccine’s transcriptomic and proteomic repertoire in cell culture is as expected. The combined transcriptomic and proteomics approaches provide a detailed insight into the behaviour of this important class of vaccine using state-of-the-art techniques and illustrate the potential of this technique to inform future viral vaccine vector design.

**Supplementary Information:**

The online version contains supplementary material available at 10.1186/s13073-021-00859-1.

## Background

Since the emergence of SARS-CoV-2 in late 2019, there has been a global effort to develop effective vaccines and at least four different adenovirus-vectored vaccines are at various stages of development or deployment [1–5]. Adenoviruses are non-enveloped viruses containing a linear double-stranded DNA genome of approximately 36 kbp that efficiently deliver their DNA genome to the nuclei of host cells for viral genome replication. Adenoviruses of mammals are in the genus *Mastadenovirus*, and these are grouped into one of 7 species (A–G) [6]. Adenovirus-based vectors typically have two regions of the virus genome removed, known as E1 and E3 [7]. The E1 region contains early genes required to trigger a transcription cascade enabling viral replication; E1-deleted vectors therefore need to be grown in E1 trans-complementing cell lines such as HEK293 cells [8]. HEK293 cells have a 4-kbp region of human adenovirus type 5 (HuAd5) integrated into the cellular genome that provides the E1 genes *in trans* enabling efficient virus vector replication and recombinant virus production. The E3 region is comprised of genes encoding proteins that primarily act to subvert the immune response to adenovirus infection and are thus not needed for replication in cell culture and potentially undesirable from a vaccine platform perspective. Usually, the transgene to be expressed is inserted into the virus genome in place of the E1 region under the control of a highly active promoter.

**Fig. 1:**
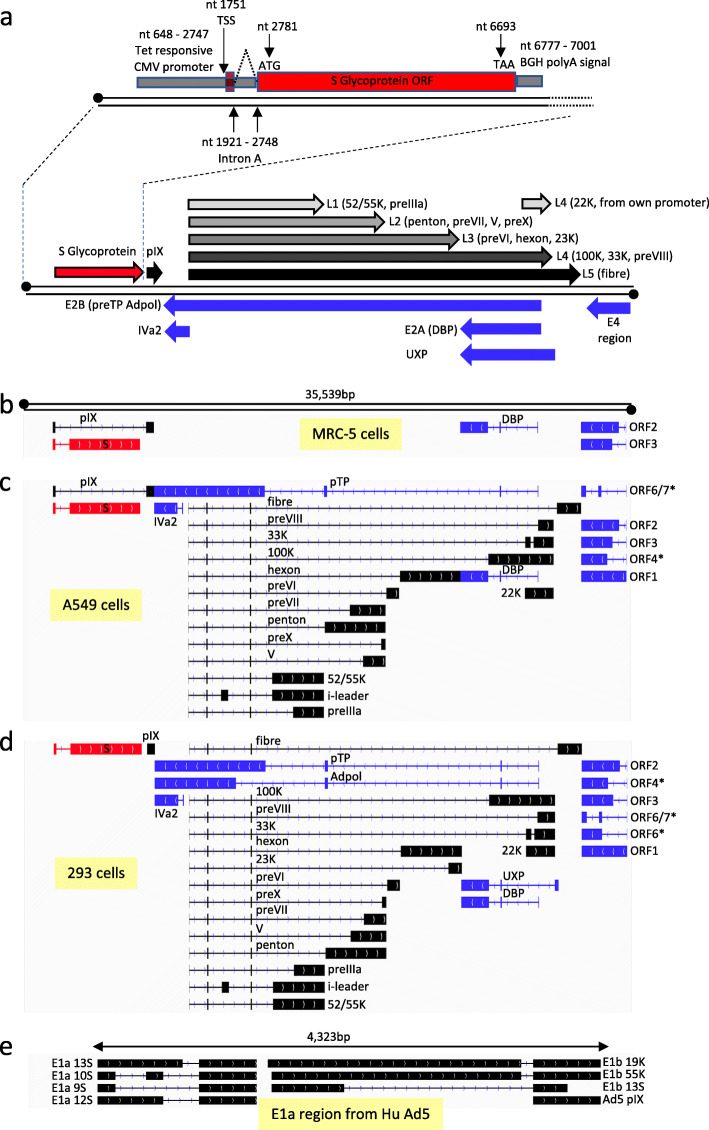
Transcription map of detected transcripts in non-permissive and permissive human cells. **a** A simplified schematic of the S glycoprotein expression cassette as inserted into the ChAdOx1 nCoV-19 vector backbone in place of the E1 region of ChAdOx1. The nucleotide positions of major features in the expression cassette are indicated. Black transcripts originate from the top strand of the dsDNA viral genome (illustrated by two black lines with circles at the 5′ ends) and blue transcripts originate from the complementary strand. Each arrow depicts the expected transcription start and polyadenylation site but omits splicing events within each transcript that give rise to mature mRNA. Most ChAdOx1 structural proteins are transcribed starting at the same promoter (known as the major late promoter) ending at one of 5 different polyadenylation sites (known as L1–L5). However, the 22K gene can also be expressed from its own promoter as shown. Typically, every member of this late transcript group has three obligate exons at the beginning known as the tripartite leader. In addition, an occasional 4th exon is included containing an ORF known as the i-leader protein. In each subsequent part (**b** to **e**), the drawing from IGV viewer illustrates the structure of the dominant transcripts expressed from the 35,539 bp ChAdOx1 nCoV-19 genome that codes for each indicated ORF as detected by dRNAseq in MRC5 cells (**b**), A549 cells (**c**) and 293 cells (**d**). For the E4 region, we have marked with an asterisk those E4 ORFs that are in fact derived from HuAd5. The rectangles indicate exons joined by lines indicating introns. In each case, the dominant transcript for the S glycoprotein of SARS-CoV-2 is indicated in red. Transcripts that map to the top strand of the virus genome are in black and those that map to the bottom strand (and are reversed in orientation) are in blue. **e** The structure of dominant transcripts derived from the 4323 bp of the HuAd5 E1 region integrated into the genome of HEK293 cells

Adenovirus-based vaccines are grown in HEK293 cells (or equivalents) to very high titres, purified and then administered to individuals. The virus will attach to a host cell, enter and deliver the recombinant genome to the nucleus where the desired gene of interest is expressed. Typically, the lack of E1-encoded proteins should ensure that there is no productive viral replication. However, it is noteworthy that removal of the E1 and E3 regions still leaves a number of other viral genes and promoters, including the E2 and E4 regions/promoters as well as the major late promoter, that normally drives expression of transcripts covering the major structural proteins of adenovirus (e.g. Fig. 1a). Moreover, background expression of at least some of these adenovirus genes has been previously identified in studies with human adenovirus-based vectors [9–12]. Expression of these viral vector genes may lead to the production of adenoviral antigens which in turn would bring the cell to the attention of the adaptive immune response [13–15]. More directly, pre-existing antibodies can be problematic, for example by neutralising the incoming vaccine vector. This has prompted the development of adenovirus vectors based on strains of adenovirus for which there is little or no pre-existing immunity in the general human population [16].

The ChAdOx1 vector virus is derived from chimpanzee adenovirus Y25 and is deleted for E1 and E3 genes. The seroprevalence of clinically relevant neutralising antibodies against Y25 in humans has been assessed in random adult serum samples and was found to be absent in UK adults (*n* = 100) compared to 9% of Gambian adults (*n* = 57) [17]. In addition, parts of the E4 region of the Y25 virus have been replaced with the equivalent E4 regions from HuAd5 to facilitate growth in HEK293 cells [17]. We have previously published data using this vaccine platform in earlier studies [18–22], and we have more recently reported on the ChAdOx1 nCoV-19 vaccine’s effectiveness in a phase 3 clinical trial [23]. In this study, we wanted to determine in detail exactly which adenovirus genes on the ChAdOx1 genome were being expressed in non-permissive cell lines alongside the S glycoprotein from SARS-CoV-2 using the latest transcriptomic and proteomic approaches. We recently developed a pipeline to analyse and characterise transcriptomic data derived from direct RNA sequencing (dRNAseq) on Oxford Nanopore devices, and we have used this pipeline to analyse the transcriptome of adenovirus [24] and SARS-CoV-2 [25] during productive viral replication. Potentially of significance for adenovirus-based vaccine delivery systems, we showed that in a wild type human adenovirus replication cycle in human cells, there is a wide range of non-standard transcripts produced as a result of numerous atypical splicing and polyadenylation events [24]. Because of this, we also wanted to understand the transcriptomic repertoire of the S glycoprotein transcript in the ChAdOx1 vector backbone. This is critical because during SARS-CoV-2 replication the S glycoprotein transcript, like all SARS-CoV-2 transcripts, is made in the cytoplasm in the absence of host splicing and polyadenylation machinery [26]. Indeed, in the ChAdOx1 nCoV-19 vector, expression of the S glycoprotein is enhanced by inclusion of an intron between the transcription start site and the S glycoprotein ORF. Inclusion of introns in gene expression cassettes is thought to promote engagement of the nascent mRNA with the host splicing, polyadenylation and mRNA export machinery and has been previously shown to significantly enhance transgene protein expression in adenoviral vectors [27]. We wanted to understand if this addition of an intron before the S glycoprotein ORF could enable unwanted splicing events. We chose to examine the transcriptomic and proteomic repertoire of ChAdOx1 nCoV-19 in two lung-derived human cell lines. MRC5 cells are genetically normal, slow growing and with a finite lifespan (although not primary), and A549 cells are immortal and cancerous. Thus, the cell types are both human and lung derived but quite distinct in their growth properties, and we reasoned this could result in distinct and informative transcriptomic expression profiles from the same vector.

We determined that transcription of the S glycoprotein gene is, as expected, by far the dominant transcript generated in both non-permissive cell lines tested. Interestingly, we noted distinct transcriptomic repertoires from the ChAdOx1 vector were possible in the two non-permissive cell lines and that at a very low-level aberrant splicing and polyadenylation of the SARS-CoV-2 S glycoprotein transcript did occur in all settings. Proteomically, we were able to detect SARS-CoV-2 S glycoprotein and several ChAdOx1 proteins as well as using phosphoproteomics to confirm that the SARS-CoV-2 S protein is phosphorylated as we have previously reported [25]. Our findings are likely to be reflected in all adenovirus E1/E3-deleted replication-defective type vectors irrespective of the parent adenovirus and represent a comprehensive and informative analysis of the transcriptomic repertoire of this important class of virus vaccine vector.

## Methods

### Virus and cells

Human MRC-5 cells (a genetically normal male human lung fibroblast-like line), A549 cells (a human male lung epithelial-like continuous line derived from carcinomatous tissue) and HEK293 cells (human embryonic kidney epithelial cell line immortalised by human adenovirus E1 region) were obtained from the European Collection of Authenticated Cell Cultures (ECACC). The cells were cultured in DMEM supplemented with 10% foetal bovine serum, 100 U/ml penicillin and 100 μg/ml streptomycin. The ChAdOx1 vector and the ChAdOx1 nCoV-19 vaccine vector have been previously reported [1, 5, 17], but briefly, the vector is derived from the Y25 chimpanzee adenovirus with E1 and E3 regions deleted and E4 region ORF 4, ORF 6 and ORF6/7 replaced with their equivalent HuAd5 counterparts to improve vector yield. The S glycoprotein ORF inserted into the vector (Genbank accession number YP_009724390.1) has been human codon optimised and contains the human tissue plasminogen activator (tPA) signal peptide at the N-terminus. Transcription of the S glycoprotein is driven by a CMV immediate promoter containing intron A [28] and the tetracycline-regulated operon sequence, and the polyadenylation signal is the bovine growth hormone (BGH) polyadenylation sequence. After reaching confluence, the cells were infected with ChAdOx1 nCoV-19 at a multiplicity of 10 infectious units per cell to ensure infections were synchronous. Duplicate flasks of infected cells were harvested at 24, 48 and 72 h post-infection (hpi) for MRC5-5 and A549 cells. One flask was used to extract total RNA whilst the duplicate was used to extract total protein content. For the HEK293 cells, just one flask was infected for total RNA extraction.

### RNA extraction and sequencing

Total RNA was extracted from the infected cells using TRIzol™ reagent (#15596026, Ambion) at 1 ml of reagent per 10^7^ cells but with two additional washes of extracted RNA with 70% ethanol prior to storage at − 80 °C under 70% ethanol. Once resuspended in H_2_O, extracted RNA was immediately enriched for polyadenylated RNA and then immediately sequenced as we have described previously for dRNAseq of human adenovirus-infected cells [24]. As before, the SQK-RNA002 kits and MIN106D R9 version flow cells (Oxford Nanopore Technologies) were used following the manufacturer’s protocols exactly. Between 0.8 and 1.5 million QC-passed reads were typically obtained per flow cell over 48 h.

### Data analysis and characterisation of viral transcripts

As before, our previously described ORF-centric data analysis pipeline was used to characterise the RNA derived from the ChAdOx1 nCoV-19 genome [24]. Briefly, the transcripts were mapped to the viral genome with minimap2 and the mapping data was used to try to identify commonly used transcription start and termination locations alongside the splice acceptor/donor sites. Once this is complete, the software then assigns each transcript to a “transcript group” depending on its pattern of transcription start, transcription termination and splice acceptor/donor sites and counts how many transcripts belong to each transcript group. This data is then used by a second in-house script to generate pseudo transcripts based on the genomic sequences alongside a table of features predicted to be present on the vector genome (e.g. ORFS and predicted transcription start sites, Additional file 1). For each transcript group, this script then determines which features (if any) are contained within this transcript group. In addition, it notes which one (if any) of the known ORFs noted in the features table are 5′ proximal for each transcript group. In this manner, transcripts with different structures that still code for the same given protein can be counted together. This script also produced GFF files describing the structure of each transcript group and also produces a GFF file describing the dominant transcript type coding for each ORF detailed in the table of features provided. Finally, the pipeline also produces a list of proteins that are 5′ proximal for any given transcript group but are not identical to the list of known proteins predicted from the features table, referred to as the “proteins not known” list.

### Characterisation of human transcripts

For each dataset, minimap2 [29] was used to map the transcripts to a list of human transcripts from Ensembl covering all human transcripts but with the fasta header modified so that each transcript included ENSG, ENST and ENSP information (Additional file 2). In this way, dRNAseq transcripts mapped to any given human transcript could be unambiguously assigned to its gene group and to the protein coded by that human transcript. An in-house script counted the number of times dRNAseq transcripts mapped to each curated Ensemble transcript and compared transcript mapping abundance between the mock and each of the time points after adjusting for the total number of mapped transcripts at each time point. This was collated as a simple table of log2 fold changes between mapping abundance of the mock sample transcripts time point and the mapping abundance of the transcripts at each time point. Further statistical evaluation was not applied since there is currently no consensus on how to use dRNAseq data to robustly infer gene expression changes or even the minimum depth of reads required [30].

### Total and phosphoproteome analysis

Protein lysates were prepared from MRC5 or A549 cells only following mock infection, or infection with ChAdOx1 nCoV-19 for 24 h, 48 h or 72 h as previously described [25].

Aliquots of 100 μg of each sample were digested with trypsin (2.5 μg trypsin per 100 μg protein; 37 °C, overnight) and labelled with Tandem Mass Tag (TMT) ten plex reagents according to the manufacturer’s protocol (Thermo Fisher Scientific, Loughborough, LE11 5RG, UK), and the labelled samples pooled according to cell line.

For the total proteome analysis, an aliquot of 50 μg of the pooled sample was desalted using a SepPak cartridge according to the manufacturer’s instructions (Waters, Milford, MA, USA). Eluate from the SepPak cartridge was evaporated to dryness and resuspended in buffer A (20 mM ammonium hydroxide, pH 10) prior to fractionation by high pH reversed-phase chromatography using an Ultimate 3000 liquid chromatography system (Thermo Fisher Scientific). In brief, the sample was loaded onto an XBridge BEH C18 Column (130 Å, 3.5 μm, 2.1 mm × 150 mm, Waters, UK) in buffer A and peptides eluted with an increasing gradient of buffer B (20 mM ammonium hydroxide in acetonitrile, pH 10) from 0 to 95% over 60 min. The resulting fractions (15 in total) were evaporated to dryness and resuspended in 1% formic acid prior to analysis by nano-LC MSMS using an Orbitrap Fusion Lumos mass spectrometer (Thermo Scientific).

For the phosphoproteome analysis, the remainder of the TMT-labelled pooled sample was also desalted using a SepPak cartridge (Waters, Milford, MA, USA). Eluate from the SepPak cartridge was evaporated to dryness and subjected to TiO_2_-based phosphopeptide enrichment according to the manufacturer’s instructions (Pierce). The flow-through and washes from the TiO_2_-based enrichment were then subjected to FeNTA-based phosphopeptide enrichment according to the manufacturer’s instructions (Pierce). The phospho-enriched samples were again evaporated to dryness and then resuspended in 1% formic acid prior to analysis by nano-LC MSMS using an Orbitrap Fusion Lumos mass spectrometer (Thermo Scientific).

High pH RP fractions (total proteome analysis) or the phospho-enriched fractions (phosphoproteome analysis) were further fractionated using an Ultimate 3000 nano-LC system in line with an Orbitrap Fusion Lumos mass spectrometer (Thermo Scientific). In brief, peptides in 1% (vol/vol) formic acid were injected onto an Acclaim PepMap C18 nano-trap column (Thermo Scientific). After washing with 0.5% (vol/vol) acetonitrile 0.1% (vol/vol) formic acid, peptides were resolved on a 250 mm × 75 μm Acclaim PepMap C18 reverse-phase analytical column (Thermo Scientific) over a 150-min organic gradient, using 7 gradient segments (1–6% solvent B over 1 min, 6–15% B over 58 min, 15–32%B over 58 min, 32–40%B over 5 min, 40–90%B over 1 min, held at 90%B for 6 min and then reduced to 1%B over 1 min) with a flow rate of 300 nl min^−1^. Solvent A was 0.1% formic acid and solvent B was aqueous 80% acetonitrile in 0.1% formic acid. Peptides were ionised by nano-electrospray ionisation at 2.0 kV using a stainless-steel emitter with an internal diameter of 30 μm (Thermo Scientific) and a capillary temperature of 300 °C.

All spectra were acquired using an Orbitrap Fusion Lumos mass spectrometer controlled by Xcalibur 3.0 software (Thermo Scientific) and operated in data-dependent acquisition mode using an SPS-MS3 workflow. FTMS1 spectra were collected at a resolution of 120,000, with an automatic gain control (AGC) target of 400,000 and a max injection time of 100 ms. Precursors were filtered with an intensity threshold of 5000, according to charge state (to include charge states 2–7) and with monoisotopic peak determination set to peptide. Previously interrogated precursors were excluded using a dynamic window (60 s ± 10 ppm). The MS2 precursors were isolated with a quadrupole isolation window of 0.7 m/z. ITMS2 spectra were collected with an AGC target of 10,000, max injection time of 70 ms and CID collision energy of 35%.

For FTMS3 analysis, the Orbitrap was operated at 30,000 resolution with an AGC target of 50,000 and a max injection time of 105 ms. Precursors were fragmented by high-energy collision dissociation (HCD) at a normalised collision energy of 60% to ensure maximal TMT reporter ion yield. Synchronous precursor selection (SPS) was enabled to include up to 5 MS2 fragment ions in the FTMS3 scan.

### Proteomics data analysis

The raw data files were processed using Proteome Discoverer software v2.1 (Thermo Scientific) and searched against a bespoke human database with the fasta headers amended to contain ENSG, ENST and ENSP notation of target human protein concatenated with a custom ChAdOx1 nCoV-19 protein database consisting of a list of “known proteins” predicted to exist by homology with known adenovirus proteins alongside the “proteins not known” list predicted by the transcriptome analysis pipeline described above (Additional file 3). Searches against this database and against an in-house “common contaminants” database were performed using the SEQUEST HT algorithm. Peptide precursor mass tolerance was set at 10 ppm, and MS/MS tolerance was set at 0.6 Da. Search criteria included oxidation of methionine (+ 15.995 Da), acetylation of the protein N-terminus (+ 42.011 Da) and methionine loss plus acetylation of the protein N-terminus (− 89.03 Da) as variable modifications and carbamidomethylation of cysteine (+ 57.021 Da) and the addition of the TMT mass tag (+ 229.163) to peptide N-termini and lysine as fixed modifications. For the phosphoproteome analysis, phosphorylation of serine, threonine and tyrosine (+ 79.966 Da) was also included as a variable modification. Searches were performed with full tryptic digestion and a maximum of 2 missed cleavages were allowed. The reverse database search option was enabled and all data was filtered to satisfy a false discovery rate (FDR) of 1%. Quantitative changes in phosphopeptide abundance were normalised with respect to total protein abundance.

### Data integration

Once the quantitative data for proteome, phosphoproteome and transcriptome were collected, an in-house script was used to integrate the data producing a seamless dataset where for any given gene, data is provided on the number of transcripts mapping to that gene at any time point alongside log2 fold changes in protein or phosphoprotein abundance (if available). For the purposes of noting changes in phosphopeptide abundance, if multiple phosphorylation sites were identified for any given protein, in the integrated dataset, the largest fold change (positive or negative) is the one reported.

## Results

### Overview of sequencing data outputs

Table 1 illustrates the number of sequence reads obtained for each timepoint and cell line combination alongside the number of reads that mapped to the human transcriptome, the ChAdOx1 nCoV-19 genome or the region of HuAd5 present in HEK293 cells. For MRC-5 cells, there was a peak of reads mapping to the ChAdOx1 genome at 48 hpi but overall the proportion of reads does not vary markedly. For A549 cells, there was a slight decline over time but notably there were approximately fivefold fewer reads as a proportion of human mapped transcripts mapping to the ChAdOx1 genome compared to MRC-5 cells. As expected, the proportion of reads that mapped to the ChAdOx1 genome in HEK293 cells, which are permissive for viral genome replication, was significantly higher. We also detected over a thousand reads mapping to the E1 and pIX region in HEK293 cells; this region covers just over 4000 bp from HuAd5 that is integrated into the HEK293 genome [31]. We utilised our previously published ORF-centric analysis pipeline to further characterise the transcripts allowing a qualitative and quantitative understanding of gene expression as we have previously done for wild type isolates of both human adenovirus type 5 and SARS-CoV-2 [24, 25]. Briefly, this analysis pipeline uses the genome sequence to correct the error-prone transcripts generated by the nanopore device. The dRNAseq data is biased towards the 3′ polyadenylated end, so we further constrained our analysis to transcripts whose mapped start site was within 50 bp of viral promoters as described for our analysis of wild type HuAd5 and SARS-CoV-2. After correction, each transcript was scanned to determine the 5′-most ORF which is compared to the locations and sequences of known ORFs and other features supplied by the user. In this way, the pipeline can identify and count the presence of known features on transcripts and ultimately provide quantitative data on the abundance of transcripts that code for each ORF. Moreover, this analysis illustrates the structure of the dominant transcript that codes for each ORF as well as characterising the structure of minor transcripts that could also code for any given ORF (Additional file 4: Table s1, Additional file 5: Table S2 and Additional file 6: Table S3).

**Table 1:** Read counts for individual dRNAseq datasets

Sample	Total reads	Longest read	Average read length	Mapped to human transcriptome	Mapped to Chadox1 nCoV-19 (E1 region in 293)	ChAdOx1 nCoV-19 mapped reads as a percentage of human mapped reads (E1 region in 293)
MRC5 mock	987,540	23,008	1607	836,380		
MRC5 24hpi	633,046	23,426	1600	578,180	6190	1.07
MRC5 48hpi	903,254	32,925	1549	810,640	13,547	1.67
MRC5 72hpi	1,020,009	26,592	1552	758,197	9723	1.28
A549 mock	1,171,427	17,654	1270	844,383		
A549 24hpi	1,411,415	22,536	1381	1,243,531	3255	0.26
A549 48hpi	1,771,635	25,111	1287	1,356,800	3194	0.24
A549 72hpi	1,790,983	19,641	1312	1,486,051	2620	0.18
293 24hpi	1,013,425	31,829	1482	768,842	109,197 (+ 1014)	14.20 (+ 0.13)

The total number of reads, longest read and average read length is noted for each dataset. In addition, the table details how many reads mapped to the human transcriptome list, the ChAdOx1 nCoV-19 genome and (in the case of HEK293 cell dataset) the E1/pIX region from human adenovirus type 5 known to be cloned into the cell line. Also shown is the percent contribution of the mapped reads relative to the total number of reads analysed. In the case of reads mapping to the ChAdOx1 nCoV-19 genome from the HEK293 cell experiment, the figures in brackets refer solely to reads mapping to the adenovirus type 5 sequences

### Comparison of ChAdOx1 nCoV-19 adenoviral gene expression levels in non-permissive and permissive cells

Strikingly, in MRC-5 cells, the ChAdOx1 vector backbone genes (i.e., excluding the S glycoprotein gene) were hardly transcribed at any timepoint, but in A549 cells, we saw a wide range of transcripts that code for almost every protein predicted to be expressed by the ChAdOx1 backbone (Fig. 1b, c and Table 2). The pattern of dominant transcripts seen in A549 cells was broadly typical of mammalian adenoviruses [32] and was similar to the expression pattern of the virus grown in permissive HEK293 cells (compare Fig. 1c, d). Looking quantitatively, Table 2 shows that in A549 cells a large number of different ChAdOx1 vector backbone genes were expressed to one degree or another with the adenovirus protein DBP being dominant. For MRC-5 cells, transcripts that code for DBP and E4 ORFs 2 and 3 could be detected, but only at 48 hpi. However in A549 cells, in addition to DBP/pTP coding E2 transcripts, transcripts that code for all the E4 ORFs except E4 ORF6 could be detected, which could explain the detectable levels of major late transcript proteins in A549 cells. Notably, ChAdOx1 vector backbone gene expression declined over the time course analysed.

**Table 2:** List of ORFs searched for and the transcript frequency for each ORF

	MRC-5 cells						A549 cells						293 cells	
Feature	Count 24 h	Percent of total at 24 h	Count 48 h	Percent of total at 48 h	Count 72 h	Percent of total at 72 h	Count 24 h	Percent of total at 24 h	Count 48 h	Percent of total at 48 h	Count 72 h	Percent of total at 72 h	Count 24 h	Percent of total at 24 h
Total of all reads	4443	100.00	9201	100.00	6849	100.00	2070	100.00	1937	100.00	1811	100.00	62,281	100.00
SARS_CoV_2_S_protein	4156	93.54	8570	93.14	6453	94.22	1536	74.20	1447	74.70	1645	90.83	10,586	17.00
None from list	148	3.33	278	3.02	214	3.12	92	4.44	112	5.78	53	2.93	4138	6.64
pIX	21	0.47	27	0.29	23	0.34	14	0.68	13	0.67	2	0.11	909	1.46
DBP(E2A)	1	0.02	3	0.03	0	0.00	205	9.90	95	4.90	13	0.72	5198	8.35
fibre(L5)	0	0.00	0	0.00	0	0.00	2	0.10	9	0.46	2	0.11	6011	9.65
hexon(L3)	0	0.00	0	0.00	0	0.00	16	0.77	20	1.03	3	0.17	3990	6.41
33K_full_length(L4)	0	0.00	0	0.00	0	0.00	10	0.48	20	1.03	7	0.39	3217	5.17
preVII(L2)	0	0.00	0	0.00	0	0.00	12	0.58	7	0.36	2	0.11	3212	5.16
100K(L4)	0	0.00	0	0.00	0	0.00	11	0.53	25	1.29	7	0.39	2212	3.55
i_leader_protein	0	0.00	0	0.00	0	0.00	18	0.87	46	2.37	11	0.61	1969	3.16
IVa2	0	0.00	0	0.00	0	0.00	6	0.29	14	0.72	3	0.17	1676	2.69
pV(L2)	0	0.00	0	0.00	0	0.00	4	0.19	3	0.15	0	0.00	1373	2.20
52/55K(L1)	0	0.00	0	0.00	0	0.00	20	0.97	28	1.45	6	0.33	1345	2.16
22K(L4)	0	0.00	0	0.00	0	0.00	9	0.43	9	0.46	2	0.11	1193	1.92
preX(L2)	0	0.00	0	0.00	0	0.00	6	0.29	8	0.41	4	0.22	1180	1.89
E4_orf2	0	0.00	2	0.02	0	0.00	0	0.00	1	0.05	2	0.11	1040	1.67
E4_orf3	0	0.00	1	0.01	0	0.00	13	0.63	8	0.41	3	0.17	1011	1.62
preVI(L3)	0	0.00	0	0.00	0	0.00	8	0.39	7	0.36	1	0.06	821	1.32
preVIII(L4)	0	0.00	0	0.00	0	0.00	1	0.05	2	0.10	1	0.06	821	1.32
penton_base(L2)	0	0.00	0	0.00	0	0.00	1	0.05	2	0.10	1	0.06	631	1.01
Ad5_E1b_19K	0	0.00	0	0.00	0	0.00	0	0.00	0	0.00	0	0.00	506	0.81
E4_orf1	0	0.00	0	0.00	0	0.00	1	0.05	0	0.00	0	0.00	286	0.46
preIIIa(L1)	0	0.00	0	0.00	0	0.00	5	0.24	3	0.15	0	0.00	239	0.38
E4_orf4	0	0.00	0	0.00	0	0.00	2	0.10	2	0.10	0	0.00	187	0.30
UXP	0	0.00	0	0.00	0	0.00	0	0.00	0	0.00	0	0.00	159	0.26
Ad5_E1a_13S	0	0.00	0	0.00	0	0.00	0	0.00	0	0.00	0	0.00	97	0.16
E4_orf6_7	0	0.00	0	0.00	0	0.00	1	0.05	0	0.00	1	0.06	79	0.13
23K_protease(L3)	0	0.00	0	0.00	0	0.00	0	0.00	0	0.00	0	0.00	70	0.11
Ad5_E1a_12S	0	0.00	0	0.00	0	0.00	0	0.00	0	0.00	0	0.00	26	0.04
preTP(E2B)	0	0.00	0	0.00	0	0.00	1	0.05	0	0.00	0	0.00	23	0.04
Ad5_pIX	0	0.00	0	0.00	0	0.00	0	0.00	0	0.00	0	0.00	10	0.02
pol(E2B)	0	0.00	0	0.00	0	0.00	0	0.00	0	0.00	0	0.00	4	0.01
Ad5_E1a_10S(171R)	0	0.00	0	0.00	0	0.00	0	0.00	0	0.00	0	0.00	3	0.00
E4_orf6	0	0.00	0	0.00	0	0.00	0	0.00	0	0.00	0	0.00	3	0.00
Ad5_E1b_13S(E1b-84R)	0	0.00	0	0.00	0	0.00	0	0.00	0	0.00	0	0.00	2	0.00
Ad5_E1b_22S(E1b 55 K)	0	0.00	0	0.00	0	0.00	0	0.00	0	0.00	0	0.00	2	0.00
Ad5_E1a_9S	0	0.00	0	0.00	0	0.00	0	0.00	0	0.00	0	0.00	1	0.00
Ad5_E1a_11S(217R)	0	0.00	0	0.00	0	0.00	0	0.00	0	0.00	0	0.00	0	0.00
Ad5_E1b_14.5S(E1b-93R)	0	0.00	0	0.00	0	0.00	0	0.00	0	0.00	0	0.00	0	0.00
Ad5_E1b_14S(E1b-156R)	0	0.00	0	0.00	0	0.00	0	0.00	0	0.00	0	0.00	0	0.00
Ad5_E1b_novel-84R	0	0.00	0	0.00	0	0.00	0	0.00	0	0.00	0	0.00	0	0.00

This table details how many transcripts would code for each indicated ORF from the ChAdOx1 nCoV-19 genome or ORFs coded by the region of Ad5 integrated into the HEK293 cell line. The “total of all reads” row indicates how many transcripts map to either the ChAdOx nCoV-19 genome or the region of human Ad5 integrated into the HEK 293 genome. The “none from list” row indicates how many transcripts did map but the pipeline was unable to correlate the 5′ most ORF on that transcript with any of the known ORFs from the genomes under consideration

Comparing the expression of ChAdOx1 gene expression in non-permissive vs permissive HEK293 cells illustrated how deletion of the E1 region affects ChAdOx1 gene expression in A549 cells where some vector backbone gene expression has occurred (Table 2). One key difference was the level of expression of the fibre (L5) transcript which was the dominant transcript in HEK293 cells (9.65% of transcripts mapped to ChAdOx1 nCoV19) after the S glycoprotein transcript but is expressed only at very low levels in A549 cells (0.46% of transcripts mapped to ChAdOx1 nCoV19 at its peak at 48 hpi, Table 2). Furthermore, transcripts that code for all the E4 region ORFs were readily detected, indicating that replacing the Y25 ORFs 4, 6 and 6/7 with their HuAd5 equivalents did not ablate the expression of the remaining E4 ORF transcripts (Fig. 1 and Table 2).

Analysis of the transcriptome of ChAdOx1 nCoV-19 in HEK293 cells also allowed the observation of transcription from the region of HuAd5 that is integrated into HEK293 cells (Fig. 1e). Notably, there is some expression of HuAd5 pIX from this region (Table 2) which was approximately 100-fold lower than the ChAdOx1-derived pIX protein. In addition, in the non-permissive cell lines, there were transcripts that code for protein IX, which appear to be derived from RNA pol II and have ignored the polyA signal after the S glycoprotein and instead used the pIX poly A signal and spliced out the coding region for S glycoprotein (Fig. 1b, c). In permissive HEK293 cells, the dominant transcript coding for pIX appeared to revert to the expected transcript for pIX with the transcription start site just upstream of the initiating AUG (Fig. 1d). Notably, the type of transcript seen in the non-permissive cells was still present in HEK293 cells, but it was no longer the dominant transcript coding for pIX (Additional file 6: Table S3).

### Expression of SARS-CoV-2 S glycoprotein transcripts

**Fig. 2:**
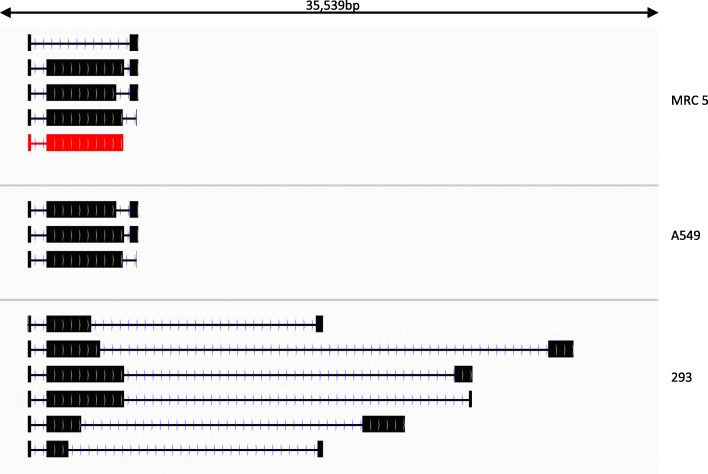
Transcript heterogeneity. This figure illustrates the structure of various transcripts observed at very low levels (less than 10 transcripts per dataset) all of which initiate at the transcription start site that drives expression of the S glycoprotein. For orientation, the structure of the dominant S glycoprotein transcript is shown in red in the examples selected from the MRC5 dataset. Three sets of example transcripts are shown from MRC5, A549 and HEK293 cells. The transcripts shown arise from splicing events that utilise only the canonical GU-AG splice donor/acceptor pair present in over 90% of eukaryotic splicing events. As can be seen, some of the transcripts arise from unintended splice site usage as well as from missing the intended BGH polyadenylation signal and utlilising other polyadenylation signals further down the vector genome

In both permissive and non-permissive cell lines, the most abundant ChAdOx1 nCoV-19 virus vector transcript, as expected, was the transcript for expression of the SARS-CoV-2 S glycoprotein. For MRC5 cells, the S transcript accounts for over 90% of transcripts from the ChAdOx1 nCoV19 genome; this is reduced to between 75 and 90% in A549 cells. However, in HEK293 cells (where there is productive viral replication), only 17% of transcripts code for the S glycoprotein relative to transcripts that code for adenovirus ORFs. This reflects the large numbers of adenovirus transcripts generated during productive replication. Notably, the S glycoprotein transcript is still the dominant transcript even in HEK293 cells. However, a number of transcripts were identified in all cell lines at low levels which appear to arise from aberrant splicing events. We also found evidence that occasionally some transcripts were spliced to other elements (notably pIX); these would still code for SARS-CoV-2 S glycoprotein but would be polycistronic (Fig. 2). We also saw limited evidence of transcripts that extended beyond the pIX polyA signal and deep into the ChAdOx1 vector backbone in HEK293 cells when there was active viral replication. However, it is important to place these rare transcripts in context—the vast majority of transcripts starting at the transcription start site for the S glycoprotein message would generate mRNA that codes for S glycoprotein (Table 3).

**Table 3:** Counts of transcripts arising from the expected transcription start site for S glycoprotein transcripts and their assignment

Cell line	Number of transcripts starting at TSS for S glycoprotein	Number of transcripts that code for S glycoprotein (percentage)	Number of transcripts belonging to the dominant transcript class (percentage)
MRC-5	20,471	19,187 (93.7%)	9746 (47.6%)
A549	4886	4607 (94.3%)	2422 (49.6%)
293	11,094	10,586 (95.4%)	6050 (54.5%)

This table details numbers of transcripts which map to the expected transcription start site for the major S glycoprotein transcript at 1700 nt. For each cell line, the counts are the accumulated numbers of transcripts at all the time points examined and the numbers (and percentage) of transcripts that should code for the S glycoprotein as the 5′ most ORF

### Additional polyadenylation site for ChAdOx1 preVI transcripts

**Fig. 3:**
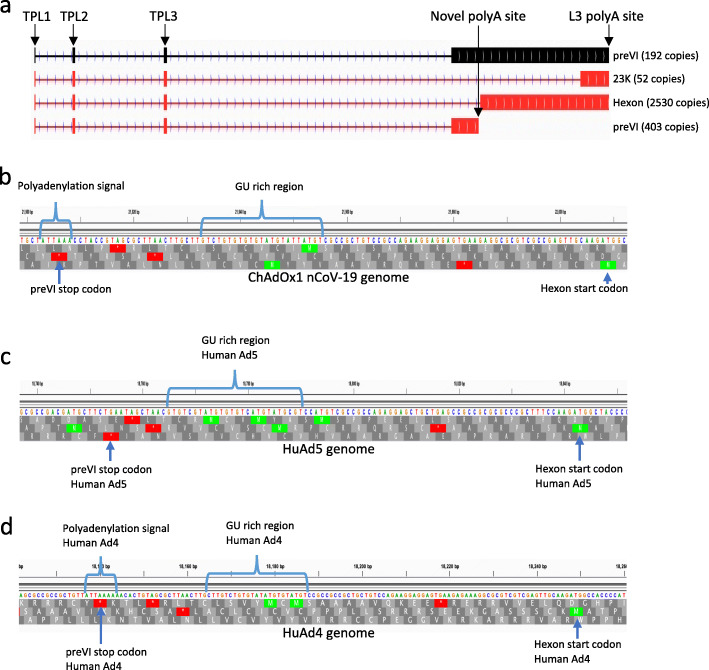
Novel polyadenylation site usage for preVI. **a** The transcript structure of transcripts coding for three proteins classically considered to be L3 proteins viz preVI, hexon and 23K proteinase. The three vertical boxes on the left represent the tripartite exons, labelled TPL1, TPL2 and TPL3, and normally included in all major late transcripts generated by mammalian adenoviruses. The L3 transcripts would classically share the same L3 polyadenylation site (indicated on the diagram). In this diagram, the dominant transcripts seen in ChAdOx1 nCoV-19-infected 293 cells for each ORF are coloured red. This figure includes, in black, the transcript structure for observed transcripts that would both code for preVI and fit the canonical transcript structure for an L3 transcript, utilising the L3 polyadenylation site. In the case of 293 cells, however, there are only 192 copies of this transcript compared to 403 copies of the novel preVI transcript indicated in red at the bottom of **a**. **b** The sequences at the proposed polyadenylation site on ChAdOx1 showing the location of the polyadenylation signal and the GU-rich region that is often present downstream of a polyadenylation signal. **c** The equivalent regions in the genome of human adenoviruses 5 (a species C adenovirus) and human adenovirus type 4 which is, like ChAdOx1, a species E adenovirus. Taken together, **b**, **c** and **d** illustrate the similarities between the two species E adenovirus genomes and their distinction from a species C adenovirus genome

Typically, in human adenoviruses, the late transcripts code for most of the structural viral proteins. The late transcripts typically originate at the major late promoter and include three exons (known as the tripartite leader) and optional i-leader exon and are grouped into five classes called L1 to L5 based on which of the five major polyadenylation sites they use [32, 33]. The transcript for preVI is usually regarded as part of the L3 group of transcripts which share a common polyadenylation site but utilise different splice acceptor/donor pairs to place one of three different ORFs (preVI, hexon or 23K) proximal to the 5′ cap for translation. In the case of ChAdOx1 replicating in HEK293 cells, although there were transcripts coding for preVI that would fit this pattern (e.g. utilising the same polyadenylation site as transcripts for hexon and 23K), the dominant transcript for preVI instead utilises an additional polyadenylation site upstream of the start codon for the hexon protein (Fig. 3a). This was also the case in A549 cells (Fig. 1b) where 12 transcripts utilised this additional polyadenylation site compared to just 4 using the classical L3 polyadenylation site (Additional file 5: Table S2). Examining the sequence of the viral genome in the region of this additional polyadenylation site reveals a GU-rich region preceded by a classical polyadenylation signal [34] (Fig. 3b)—this signal is not present in the equivalent region of HuAd5 (Fig. 3c) where no such preVI-specific polyadenylation site has been observed [24]. However, this additional polyadenylation site is present in human adenovirus type 4 (HuAd4, Fig. 3c) which is also a member of the group E mammalian adenoviruses like chimpanzee adenovirus Y25 from which ChaAdOx1 was derived.

### Proteomic detection of SARS-CoV-2 S glycoprotein and ChAdOx1 proteins

As expected, we were able to detect a range of S glycoprotein peptides in MRC5 and A549 cells as well as one phosphorylation site (Ser 1292) derived from the C-terminal portion of the glycoprotein which is internal to the viral particle (Table 4 and Additional files 7, 8, 9, 10, 11, 12, 13, 14: Tables S4, S5, S6, S7, S8, S9, S10, S11). For the vector backbone expressed proteins, we were only able to detect E4ORF3 in MRC5-infected cells whereas in A549 cells in addition to E4ORF3 we also detected DBP and hexon (Table 4). Interestingly in both MRC5 and A549 cells, we detected a peptide (SYLTPGDSSSGWTAGAAAY, aa248-265) apparently derived from a single transcript designated mRNA#962. However, this transcript has only one copy present in the transcriptome of the A549-infected cells and a similar transcript was not detected in the MRC-5 cells. Alternatively, we believe it is more likely that the detected peptide resulted from proteolytic activity at aa265 on full-length S glycoprotein either from intracellular processes or during sample harvest and preparation. In support of this, an analysis of the proteomics data to include semi tryptic peptides did identify the additional peptide (i.e. on the C-terminal side of an expected cleavage at aa265) which would be consistent with proteolytic activity at this site. The list of possible proteins predicted by translation of the 5′-most ORF of all the observed transcripts is very large and varied, and they were all used in the initial searches for peptides. However, this peptide was the only one that could not be predicted by the standard list of expected ChAdOx1 proteins. The proteomic analysis supported the transcriptomic analysis suggesting that the ChAdOx1 nCoV-19 vaccine does not make additional unexpected proteins. Notably, in both A549 and MRC5 cells, we observed some of the highest fold increases over time for the S glycoprotein as expected (Additional file 7: Table S4 and Additional file 9: Table S6).

**Table 4:** Peptides identified as derived from ChAdOx1 nCoV-19 in each cell line used

	A549 total proteome analysis—number of unique peptides identified	A549 phosphoproteome analysis—number of unique phospho sites identified	MRC5 total proteome analysis—number of unique peptides identified	MRC5 phosphoproteome analysis—number of unique phospho sites identified
SARS-CoV-2 S glycoprotein	39	1	43	1
ChAdv DBP	16	4	ND	ND
ChAdv Hexon	2	ND	ND	ND
ChAdv E4 ORF3	3	ND	2	ND
mRNA#962	1	ND	1	ND

The table lists the number of unique peptides identified and unique phosphorylation sites identified for the proteins listed. ND indicates that none was detected in that sample, ChAdv indicates a protein from the ChAdOx1 viral vector backbone and mRNA#962 indicates the truncated S glycoprotein coded by this single transcript

### Analysis of the cellular total transcriptome, proteome and phosphoproteome

Whilst our primary focus was on the transcriptome, proteome and phosphoproteome of the ChAdOx1 nCoV-19 vaccine, we also collected data on the host cell counterparts which are summarised in Additional file 15: Table S12 and Additional file 16: Table S13. The two cell lines appear to have distinct patterns of response to the vaccine virus. We collated lists of proteins that were increased or decreased in abundance at least twofold at each time point and utilised STRING pathway analysis to determine if cellular pathways were apparently overrepresented (Additional file 15: Table S12 and Additional file 16: Table S13). Whilst the host cell responses to the vaccine are varied, our STRING analysis suggested that in MRC-5 cells there was an apparent enrichment for upregulated proteins involved in the unfolded protein response (e.g. HSPA6 and SLC5A3) and ER stress (e.g. HSPA5, DNAJB9 and HERPUD1), which was not the case for A549 cells. In A549 cells, there was some evidence that ChAdOx1 nCoV-19 infection affected proteins involved in ribosome biogenesis and host mRNA splicing (lists of proteins analysed are detailed in Additional file 15: Table S12 and Additional file 16: Table S13).

## Discussion

Recombinant adenovirus vaccines have been developed over many years and have shown great promise as safe and effective vaccine platforms for mass vaccination programmes. Despite their widespread development, this is the first study to directly and comprehensively survey the transcriptomic repertoire of a replication-defective adenovirus in a non-permissive host cell. A significant advantage of using simian-based adenoviruses like ChAdOx1 (a species E non-human adenovirus) to study the transcriptome of E1-deleted adenoviruses is the absence of replication-competent adenoviruses (RCAs) [35]. RCAs can arise from homology between the HuAd5 (a species C human adenovirus) sequences present in the HEK293 cells used to produce the vaccine vector which extend beyond the E1 region into the pIX gene (as shown in Fig. 1e) and sequences present in the recombinant vector itself (e.g. pIX gene in the vector), which are frequently based on HuAd5. Whilst the emergence of an RCA could confound such an analysis in other adenoviral backgrounds, we have never detected RCA in any preparation of ChAdOx1-based vaccine vectors. This is presumably due to insufficient homology between the species E chimpanzee adenoviruses and the sequences in HEK293 cells [36]. Given the significant advantages in using simian-based vectors as a human vaccine platform [37], we wanted to deepen our understanding of these virus vectors on a molecular level. We were especially keen to determine that no additional unanticipated transcripts or proteins were being made. Any one of such proteins could be antigenic with unintended consequences (e.g. generating auto-immune responses for example).

The two host cell lines chosen for this study have distinct properties despite both being derived from male human lungs. The A549 cell line is an immortal cancerous cell line with an average of 66 chromosomes and a deletion in the CDKN2A locus leaving the cell line defective in the p53/ARFp14/MDM2 pathway. By contrast, MRC5 cells are genetically normal with a finite cell passage capability. Thus, the distinct transcriptional profiles seen from the ChAdOx1 nCoV-19 vaccine could result from the A549 cells being genetically defective and immortal or it may be connected to unrelated differences in the intracellular environment. Previous work with human adenovirus-based vectors has suggested that a cellular E1A-like activity is in some manner connected to pre-existing levels of heat shock proteins, with promyelocytic leukaemia protein (PML) also being implicated [38, 39]. Notably, in the mock transcriptomic data, there are higher levels of sequenced HSPA1A and HSPA1B transcripts in A549 cells compared to MRC-5 cells prior to infection with ChAdOx1 nCoV-19 with the reverse being true for PML transcripts (Additional file 15: Table S12 and Additional file 16: Table S13). We noted that in MRC5 cells, levels of transcripts for HSPA1A and HSPA1B show increases over time and for HSPA1B there is a corresponding protein increase (Additional file 15: Table S12 and Additional file 16: Table S13), whereas in A549 cells, there is a decrease in sequenced transcripts for HSPA1A and HSPA1B with no significant change in protein levels for HSPA1A. This supports previous research which implies heat shock (and thus increased expression of heat shock proteins) does not overcome the lack of E1A expression [39] as in MRC5 cells we do not see increasing amounts of ChAdOx1 vector backbone transcription. In addition, NFκB has been implicated in enabling HuAd5-based vector backbone expression in non-permissive cell lines [40]. However, no marked differences were observed between the two cell lines in the transcriptomic abundance data for NFKB1 in our datasets (Additional file 15: Table S12 and Additional file 16: Table S13). A deeper understanding of the intracellular environments that allow the ChAdOx1 vector to overcome the lack of E1A, even in a limited fashion, could lead to better ways to prevent this which in turn may lead to improved transgene expression. The transcriptome and proteome of the two cell lines appeared to respond differently to the vaccine vector. However, whether these differences are driven by or a result of the distinct adenovirus vector expression patterns observed on the vector genome is yet to be determined.

That we observe quite distinct vaccine backbone expression in cell lines from the same tissue site is notable. However, care is needed not to overinterpret this study which was designed to investigate the potential repertoire of transcription from a respiratory virus-based vaccine vector in two different human respiratory cell lines. This vaccine vector is currently administered intramuscularly (other routes of administration including respiratory ones may also prove effective longer term), and so in further analysis, it might be useful to examine primary cells and/or cell lines derived from muscles or even biopsy material. In addition, there is clearly the potential for other cell types to become infected after intramuscular administration that may also be relevant to the adaptive immune response.

We have previously analysed the proteome of purified adenovirus particles, both wild type HuAd5- and HuAd5-based recombinant vaccine vectors, including a sample of adenovirus manufactured to clinical grade. We were able to detect non-structural proteins DBP, 100K and E4 14.7K protein in purified virus particles in addition to the expected viral structural proteins [41]. If any cells in a vaccinee did express the full range of adenovirus proteins over time similar to the A549 transcriptomic profile, then T-cell responses to the virus vector could both derive from the incoming viral proteins and from subsequent low-level expression of any of the remaining virus vector backbone genes. That some cells allow low-level expression of ChAdOx1 vector backbone transcripts would be consistent with data from human adenovirus vector studies where additional deletions in E2 and E4 or helper-dependent adenovirus vectors (where essentially all the vector backbone genes are removed) were shown to afford longer transgene expression in vivo [13, 15, 42–44]. The continuing low-level expression of vector backbone genes is therefore likely to be the main driver of immune-mediated clearance of cells infected with E1/E3-deleted adenoviruses. The repercussions in a vaccinee of broad low-level expression as observed in A549 cells is difficult to evaluate at this stage, but in principle, it suggests that repeated re-administration of a particular vaccine vector beyond the usual prime-boost vaccination regimen should be examined in further detail. In addition, low-level expression of vector backbone genes in A549 cells may be related to our finding that A549 cells express lower levels of S glycoprotein mRNA as a proportion of total mRNA than MRC5 cells (Table 1).

Our proteomics analysis is consistent with the transcriptomics data in that the S glycoprotein is readily detected by MS/MS and that we detect a slightly wider array of ChAdOx1 proteins in A549 cells compared to MRC-5 cells. We also detect evidence of phosphorylation of the S glycoprotein as we have noted previously in our work on SARS-CoV-2 [25]; the significance of this phosphorylation is unclear at this time. We do not detect any evidence of additional unexpected proteins being expressed by the ChAdOx1 nCoV19 vaccine despite a broad search of proteins coded for by all transcripts that map to the ChAdOx1 nCoV-19 genome.

We previously analysed the transcriptomic repertoire of wild type HuAd5 in MRC-5 cells showing that during a productive infection there is significant low-level heterogeneity in the usage of both splice sites and polyadenylation signals [24]. Here, we show that as with the human virus there is similar low-level expression of transcripts with aberrant splice site and polyadenylation signal usage in cell lines infected with the recombinant ChAdOx1 nCoV-19 virus in replication permissive and non-permissive cell lines. Critically, the overwhelming majority of S glycoprotein transcripts have the expected structure with only a small minority of transcripts originating from the S glycoprotein transcription start site being unable to express the S glycoprotein. The most common issue seems to be failure to use the polyadenylation signal immediately after the S glycoprotein ORF. This leads to, for example, usage of the pIX polyadenylation site and the opportunity for splicing events that may disrupt S glycoprotein expression. Other S transcripts with aberrant splice patterns or polyadenylation site usage were detected, but these were minor compared to the dominant transcript and we did not observe peptides that could arise from such aberrant transcripts. In future vector designs, this approach should highlight such issues potentially allowing changes to vector design to minimise issues identified.

Notably, in our analysis of wild type HuAd5 transcripts, we proposed that a key aspect of HuAd5 evolution would be the exploration of different splice acceptor/donor sites and initiating codon combinations. Here, analysis of expression from the ChAdOx1 backbone identified alternative usage of polyadenylation signals. Typically, the polyadenylation signal for the preVI protein transcript would be shared with the transcripts for hexon and 23K—collectively known as the L3 group since they share this polyadenylation signal. Protein preVI uses two polyadenylation signals, one that is the L3 location used by the hexon and 23K transcripts and a second just after its own stop codon. That this second one is not observed in human adenovirus type 5 is likely due to the lack of a strong polyadenylation signal as shown in Fig. 3. However, this arrangement seems to be present both in HuAd4 and chimpanzee adenovirus Y25 (which the ChAdOx1 vector is based on) which are both species E adenoviruses. Indeed, HuAd4 is closely enough related to chimpanzee adenoviruses to suggest that the human virus may have jumped into chimpanzees at some point in the past [45]. What advantage, if any, the two polyadenylation sites offer the ChAdOx1 virus is not clear, but the use of the polyadenylation site after the preVI ORF would by necessity reduce the influence of splice site selection in determining whether preVI, hexon or 23K would be expressed from transcripts utilising the canonical L3 polyadenylation site. This could, in principle, bias gene expression in favour of preVI relative to hexon or 23K. Whether this additional polyadenylation site is being selected for or against remains an open question.

Examination of the HEK293 cell data revealed, as expected, expression of HuAd5 transcripts corresponding to E1 region genes dominated by E1b19K, E1a12S and E1a13S as we saw for wild type HuAd5 gene expression at 16hpi infection [24]. As noted in the results, in HEK293 cells, we observed a small number of HuAd5 pIX transcripts, approximately 100-fold fewer than ChadOx1 pIX transcripts. However, with 240 copies of protein IX per virus capsid [46–48], it is possible that the ChAdOx1 virus particles grown in HEK293 cells (or derivatives) contain one or two copies of HuAd5-pIX per viral particle assuming the two proteins are equivalently interchangeable on the virus particle.

## Conclusions

Our analyses provide valuable insight into the transcriptomic and proteomic repertoire of an important vaccine, providing confirmation that the vaccine vector’s transcriptome is essentially as intended in these cell lines. However, care should be taken when extrapolating the results of the transcriptomic analysis using single cell types to the complex multicellular differentiated cell-rich environment these types of vaccines encounter when they are administered (by whatever route). Recently, an in-depth multi-omic analysis of the herpes virus genome revealed that an oncolytic herpes virus licenced in 2015 under the name Imlygic is in fact deleted for an additional third gene rather than the two intended because of a previously unknown ORF present in the deleted region [49]. Whilst there is no suggestion that this has been a problem, it highlights the importance of utilising state-of-the-art and unbiased approaches to survey genetically modified viruses intended for clinical use. Finally, we argue that this kind of analysis is relatively straightforward and should be routinely incorporated into the early stages of future viral vector evaluation pipelines to allow a robust understanding of the transcriptomic potential of engineered viral vectors.

## Supplementary Information

**Additional file 1.** Features file. A text file delimiting the locations of known features on the ChAdOx1 nCoV-19 genome.

**Additional file 2.** Known human transcripts. A fasta file of human transcripts from the EBI data repository and used for mapping of sequence data to the human transcriptome.

**Additional file 3.** Proteins search list. The protein search list used in the proteomics analysis.

**Additional file 4: Table S1.** MRC5 transcript groups. An excel spreadsheet describing the structure of all ChAdOx1 nCoV19 derived transcript groups identified and how many transcripts belong to each group. In addition, it also describes what features are present on each transcript group. This list is the combined data from all time points in MRC5 cells.

**Additional file 5: Table S2.** A549 transcript groups. An excel spreadsheet describing the structure of all ChAdOx1 nCoV19 derived transcript groups identified and how many transcripts belong to each group. In addition, it also describes what features are present on each transcript group. This list is the combined data from all time points in A549 cells.

**Additional file 6: Table S3.** HEK293 transcript groups. An excel spreadsheet describing the structure of all ChAdOx1 nCoV19 derived transcript groups identified and how many transcripts belong to each group. In addition, it also describes what features are present on each transcript group. This list is the combined data from all time points in 293 cells.

**Additional file 7: Table S4.** A549 proteins identified. List of all proteins identified in A549 cells or A549 cells infected for various times with ChAdOx1 nCoV-19.

**Additional file 8: Table S5.** A549 phosphoproteins identified. List of all phosphoproteins identified in A549 cells or A549 cells infected for various times with ChAdOx1 nCoV-19.

**Additional file 9: Table S6.** MRC5 proteins identified. List of all proteins identified in MRC5 cells or MRC5 cells infected for various times with ChAdOx1 nCoV-19.

**Additional file 10: Table S7.** MRC5 phosphoproteins identified. List of all phosphoproteins identified in MRC5 cells or MRC5 cells infected for various times with ChAdOx1 nCoV-19.

**Additional file 11: Table S8.** A549 Peptide Spectral Matches. List of all peptide spectral matches (PSMs) identified in A549 cells or A549 cells infected for various times with ChAdOx1 nCoV-19.

**Additional file 12: Table S9.** A549 phospho Peptide Spectral Matches. List of all phospho peptide spectral matches (PSMs) identified in A549 cells or A549 cells infected for various times with ChAdOx1 nCoV-19.

**Additional file 13: Table S10.** MRC5 Peptide Spectral Matches. List of all peptide spectral matches (PSMs) identified in MRC5 cells or MRC5 cells infected for various times with ChAdOx1 nCoV-19.

**Additional file 14: Table S11.** MRC5 phospho Peptide Spectral Matches. List of all phospho peptide spectral matches (PSMs) identified in MRC5 cells or MRC5 cells infected for various times with ChAdOx1 nCoV-19.

**Additional file 15: Table S12.** Integrated transcriptomics and proteomics table, A549 cells. Integrated list of transcriptomic, proteomic and phosphoproteomic changes over time in A549 cells.

**Additional file 16: Table S13.** Integrated transcriptomics and proteomics table, MRC5 cells. Integrated list of transcriptomic, proteomic and phosphoproteomic changes over time in MRC5 cells.

## Data Availability

The mass spectrometry proteomics data have been deposited to the ProteomeXchange Consortium (http://proteomecentral.proteomexchange.org) via the PRIDE partner repository [50] with the dataset identifier PXD023286, https://www.ebi.ac.uk/pride/archive/projects/PXD023286 [51]. All the fastq files are available from the Short Read Archive with the identifier PRJNA688412, https://www.ncbi.nlm.nih.gov/sra/PRJNA688412 [52].
